# Does Environmental Information Disclosure Improve the Health Level of Middle-Aged and Old Residents? Evidence From China

**DOI:** 10.3389/fpubh.2022.776850

**Published:** 2022-03-17

**Authors:** Xuan Yu, Weiteng Shen, Sen Lin

**Affiliations:** ^1^Business School, Ningbo University, Ningbo, China; ^2^Donghai Strategic Research Institute, Ningbo University, Ningbo, China; ^3^Business School, Zhejiang Wanli University, Ningbo, China; ^4^Ningbo China Institute for Supply Chain Innovation, Ningbo, China; ^5^Nottingham University Business School China, University of Nottingham Ningbo China, Ningbo, China

**Keywords:** environmental information disclosure, health level, middle-aged and old residents, impact analysis, empirical research

## Abstract

**Objective:**

The purpose of this study is to empirically examine the impact of environmental information disclosure on the health of middle-aged and old residents and investigate whether such disclosure can improve the health of middle-aged and old residents.

**Methods:**

This study matches the data of the Pollution Information Transparency Index (PITI) and China Health and Retirement Longitudinal Study in 2018 and uses the ordered logistic regression model to assess the impact of environmental information disclosure on the health of middle-aged and old residents. Furthermore, stepwise regression, ordinary least square, and ordered probit regression models are used for robustness tests. The IV-Ordered probit regression model solves the endogenous problem.

**Results:**

Environmental information disclosure has a significant positive correlation with the health level of middle-aged and old residents. After the robustness test and endogenous problem handling, this conclusion still holds. Estimation results show that when PITI increases by 1 unit, the probability of improving the self-reported health level and actual health level of middle-aged and old residents increases by 1 and 0.87%, respectively. The impact of environmental information disclosure on the health of middle-aged and old residents also has significant regional heterogeneity. Specifically, the impact is mainly reflected in the central region of China.

**Conclusion:**

Environmental information disclosure can improve the health of middle-aged and old residents. To improve the health of middle-aged and old residents, it is necessary to implement and enhance the environmental information disclosure system continuously. The anti-driving effect of environmental information disclosure on the treatment of environmental pollution must be intensified further, particularly focusing on the central region of China, where is more polluted and more concentrated than other regions.

## Introduction

Population aging is an important issue currently facing China (1–3). According to the results of China's seventh national census (4), as of November 2020, China has a population of 264,018,766 people aged 60 and over, accounting for 18.7% of the total population and far exceeding the 10% specified in internationally accepted aging standards. There are 190,635,280 people aged 65 and over, accounting for 13.50% of the total population and far more than the 7% stipulated in international standards. Furthermore, China's aging population is increasing. Compared with the sixth national census in 2010, the proportion of people aged 15–59 in the seventh national census in 2020 dropped by 6.79%, but the proportion of the population aged 60 and 65 years and above increased by 5.44 and 4.63%, respectively (5). In addition to the ever-increasing total number of middle-aged and old people, China's middle-aged and old residents are also plagued by chronic diseases (6, 7). According to official Chinese statistics (8), in 2018, the prevalence of chronic diseases among residents over 45 years old in China was 47.32%, while the prevalence of chronic diseases among residents aged 65 and over was as high as 62.33%. The high proportion of the middle-aged and old population and the high prevalence of chronic diseases, coupled with China's low-level doctor-patient ratio (9), have further aggravated medical pressure in the country and hindered its social and economic development (10, 11).

Numerous studies have confirmed the close relationship between the ecological environment and the health of residents (12–14), especially in terms of middle-aged and old residents (15–17). China's rapid economic development has significantly improved the living standards of such residents. However, China's economic growth has been characterized by high energy consumption, high pollution, and high emissions (18) since the reform and opening-up, which has brought various ecological and environmental issues such as smog pollution, which is well-known to the public (19), industrial pollution (20), water pollution (21), and offshore pollution (22). These environmental pollution problems have caused great harm to the health of residents. Kampa and Castanas (23) believes that air pollution has both acute and chronic effects on human health, affecting a significant number of different systems and organs, including heart disease, lung cancer, acute respiratory infections in children and chronic bronchitis in adults. Mannucci and Franchini (24) indicates that at least seven million deaths globally are attributable to the effects of air pollution annually. Ashbolt (25) and Schwarzenbach et al. (26) believe that drinking water is a major source of microbial pathogens in developing regions. Wang and Yang (27) studies the relationship between health and water pollution using the random-effects and random effects logit models. He demonstrates that the negative health effects of water pollution remain a major source of morbidity and mortality in China.

To control environmental pollution, the Chinese government has issued a large number of policies, including environmental information disclosure. Environmental information disclosure is an environmental governance policy based on information disclosure, which is regarded as a supplement to administrative and market means (28–30). Since the “*Environmental Protection Law of the People's Republic of China*” put forward the concept of environmental information disclosure in 1989, such disclosure in China has experienced more than 30 years of practice and development, and the related policy has been gradually improved through continuous adjustments and changes (31). If we advance the time to before the reform and opening up, China's environmental information disclosure system has even gone through about 70 years of construction and exploration, forming a circular circle from system formulation, implementation to amendment. Before the reform and opening up, environmental information in China was an implicit disclosure. After the 1990s, China's environmental information entered a formal disclosure phase. After entering the 21st century, environmental information began to be disclosed centrally and a series of supporting policies began to be released, including the “*Measures for Disclosure of Environmental Information*,” “*Application for Disclosure of Environmental Information According to Law*,” “*Measures for Reporting Environmental Emergency Information*,” and “*Measures for Disclosure of Environmental Information by Enterprises and Institutions*.” As a policy tool, environmental information disclosure has had a significant positive impact on improving China's ecological environment (32–35).

At present, scholars have studied the relationship between environmental pollution and the health of middle-aged and old residents as well as the relationship between environmental information disclosure and environmental pollution. However, they rarely combine the two to study the impact of environmental information disclosure on the health of middle-aged and old residents. In terms of macro policy, the Chinese government has also placed greater emphasis on environmental and health issues. In the 14th Five-Year Plan released in March 2021, it is proposed to comprehensively promote the construction of “Healthy China,” and set a goal to increase life expectancy per capita by 1 year during the 14th Five-Year Plan. At the 75th session of the United Nations General Assembly, Chinese President Xi Jinping directly announced China's “dual carbon” goal, which is to peak CO_2_ emissions by 2030 and work toward achieving carbon neutrality by 2060. In conclusion, it is of great theoretical and practical significance to study the relationship between environmental information disclosure and the health level of middle-aged and old residents.

This study matches the environmental information disclosure indicators at the urban level in China with the micro-survey data of middle-aged and old residents. It uses the ordered logistic regression model to study the impact of environmental information disclosure on the health of middle-aged and old residents. We find that environmental information disclosure can improve the health level of middle-aged and old residents. After the robustness test and endogenous problem handling, this conclusion still holds. We also find significant regional differences in the impact of environmental information disclosure on the health of middle-aged and old residents, and this impact is mainly reflected in the central region of China. The marginal contribution of this study is that we provide a new perspective for studying the impact of environmental policies on health, that is, environmental information disclosure system. From the perspective of environment and health, the implementation of the environmental information disclosure system is not only conducive to solving the problem of environmental pollution, but also has a positive impact on the improvement of residents' health. These results provide more evidence to government to development and implement evidence-based strategies and policies to improve the residents' health.

The remainder of this study is organized as follows. Section Materials and Methods introduces the materials and methods, including data sources, specification of variables and ordered logistic regression model. Section Results presents the results of the research, which used Stata, including tests of parallel regression assumption, benchmark regression, robustness test, endogenous problem handling, and regional differences. Section Discussion discusses the empirical results based on previous studies. Finally, Section Conclusion provides a summary of the results and puts forward some policy recommendations and the direction of further research.

## Materials and Methods

### Data Sources

The data source of this article includes two parts, with the most important one being the China Health and Retirement Longitudinal Study in 2018 (CHARLS-2018) hosted by the National School of Development of Beijing University. CHARLS is a longitudinal survey that aims to be representative of the residents in mainland China aged 45 and older, with no upper age limit. It attempts to set up a high-quality public micro-database that can provide a wide range of information from socioeconomic status to health conditions to serve scientific research needs or the elderly (36). Currently, CHARLS is widely used in the study of the health of middle-aged and old people in China (37–40). The national baseline survey was conducted in 2011, with wave 2 in 2013, wave 3 in 2015, and wave 4 in 2018, respectively. To ensure sample representativeness, the CHARLS baseline survey covered 28 provinces, 150 countries/districts, and 450 villages/urban communities across the country, reflecting the middle-aged and older Chinese population collectively. The sample of CHARLS-2018 involved ~19,000 individuals in 12,400 households. Data of CHARLS-2018 are available at http://charls.pku.edu.cn/.

Another source of data is the Pollution Information Transparency Index (PITI), which is co-developed by the Institute of Public and Environmental Affair (IPE) and The Natural Resources Defense Council. At present, many studies use PITI to represent the environmental information disclosure of the region (41–43). Matching with CHARLS-2018, this article selects the PITI of 2018, covering 29 provinces and 120 cities in China. Data and more information about PITI are available at http://www.ipe.org.cn/.

### Specification of Variables

#### Explained Variables

The explained variable of this article is the health level of middle-aged and old residents, which is divided into self-reported health level (*self_HL*) and actual health level (*actual_HL*). Self-reported health level is an individual's subjective judgment of their health. Using it to characterize health status can meet the needs of analyzing the relationship between environment and health to a large extent (44). Self-reported health level can be obtained through a question in the CHARLS-2018 questionnaire:

- What do you think of your health?

In the questionnaire, the answer to this question is divided into five levels: 1 = very good, 2 = good, 3 = fair, 4 = poor, and 5 = very poor. To be consistent with the direction of the explanatory variables (the larger the value, the better), we re-encoded the level of this question to 1 = very poor, 2 = poor, 3 = fair, 4 = good, and 5 = very good.

The actual health level needs to be determined through a series of tests. This article selects a series of physical functional limitations in the questionnaire to be investigated. These questions include:

- Do you have any difficulty with running or jogging about 1 km?
- Do you have difficulty with getting up from a chair after sitting for a long period?
- Do you have difficulty with climbing several flights of stairs without resting?
- Do you have difficulty with stooping, kneeling, or crouching?
- Do you have difficulty with reaching or extending your arms above shoulder level?
- Do you have difficulty with lifting or carrying weights over 5 kg, such as a heavy bag of groceries?
- Do you have difficulty with picking up a small coin from a table?

In the questionnaire, these seven questions adopt a four-level evaluation standard: 1 = No, I don't have any difficulty, 2 = I have difficulty but can still do it, 3 = Yes, I have difficulty and need help, and 4 = I can't do it. We add up the total scores of these seven questions and measure the actual health level of residents according to Table 1. The higher the total score, the lower the actual health level. As with the self-rated health level, we also coded these levels as follows: 1 = very poor, 2 = poor, 3 = fair, and 4 = good.

**Table 1:** Evaluation standard of *actual_HL*.

**Score**	**Score = 7**	**7 < score ≤ 14**	**14 < score ≤ 21**	**21 < score ≤ 28**
*actual_HL*	Good	Fair	Poor	Very poor

#### Explanatory Variables

The explanatory variable of this article is the level of environmental information disclosure, measured by PITI. The full score of PITI is 100. The higher the score, the higher the level of environmental information disclosure in the region. PITI's accounting is divided into five dimensions: regulatory information, self-monitoring, interactive response, emissions data, and environmental impact assessment (EIA) information, including eight indicators. Table 2 shows the specific description and weight of each indicator.

**Table 2:** Accounting standards of PITI.

**Dimensions**	**Indicators**	**Weights (%)**
Regulatory information	Daily violation records that exceed the standard	25
	Evaluation of corporate environmental behavior	5
Self-monitoring	Automatic monitoring of state-controlled enterprises	20
	Key polluting enterprises	6
Interactive response	Environmental protection inspectors and complaints	7
	Disclosure based on application	8
Emissions data	Disclosure of corporate emissions data	14
EIA Information	Disclosure of EIA Information	15

*Source of information: The 2018-2019 Annual Pollution Information Transparency Index Assessment*.

#### Control Variables

To improve the accuracy of the model estimation results, we refer to some literature that studies the factors affecting the health of middle-aged and old residents. Based on the availability of data, we selected some factors as control variables and put them in the model. There are five dimensions of control variables: (1) Demographic characteristics, including gender (*gender*), age (*age*), education level (*edu*), and marriage status (*marriage*); (2) Living habits, including sleep status (*sleep*), whether the individual smokes (*smoke*), frequency of drinking (*drinking*), and whether physical activity is performed (*physic*); (3) Medical behaviors, including whether to participate in medical insurance (*insurance*) and medical and fitness expenditures (*health_ep*); (4) Health level in childhood (*health_ch*), that is, the health status before the age of 15; and (5) Urban or rural residents (*urban*). It is worth noting that unhealthy diet is also an important risk factor affecting the health level of middle-aged and old residents. However, there was no survey related to dietary habits in the CHARLS-2018 questionnaire; therefore, the control variables in the model do not include this factor. Table 3 shows the specific description and value meaning of all variables.

**Table 3:** Variable definitions.

**Types**	**Dimensions**	**Symbol**	**Remarks**
Explained variables	Health level of middle-aged and old residents	*self_HL*	1 = Very Poor, 2 = Poor, 3 = Fair, 4 = Good, 5 = Very good
		*actual_HL*	1 = Very Poor, 2 = Poor, 3 = Fair, 4 = Good
Explanatory variables	Degree of environmental information disclosure	*PITI*	–
Control variables	Demographic characteristics	*gender*	1 = Male, 2 = Famale
		*age*	–
		*edu*	Value range 1–11^a^
		*marriage*	1 = Married, 0 = Never married/Separated/Divorced/Widowed
	Living habits	*sleep*	During last month average hours of actual sleep
		*smoke*	1 = Yes, 0 = No
		*drinking*	1 = Drink more than once a month, 2 = Drink but less than once a month, 3 = Don't drink
		*physic*	1 = Yes, 0 = No
	Medical behavior	*insurance*	1 = Yes, 0 = No
		*health_ep*	Unit: 1,000 Yuan
	Health level in childhood^b^	*health_ch*	1 = Poor, 2 = Fair, 3 = Good, 4 = Very good, 5 = Excellent
	Urban or rural residents	*urban*	1 = Urban, 0 = Rural

a *1 = No formal education, 2 = Did not finish primary school, 3 = Sishu/home school, 4 = Elementary school, 5 = Middle school, 6 = High school, 7 = Vocational school, 8 = 2-/3-Year College/Associate degree, 9 = 4-Year College/Bachelor's degree, 10 = Master's degree, 11 = Doctoral degree/Ph.D*.

b *Childhood means before the age of 15*.

Table 4 shows the descriptive statistical results of all variables. The second column indicates the number of observations (Obs) for each variable. PITI matches approximately half of the CHARLS-2018 sample. The mean, standard deviation (SD), minimum (Min), and maximum (Max) can reflect the measures of dispersion of the sample. To test whether multicollinearity exists between variables, we calculate the variance inflation factor (VIF) of the explanatory variable and all control variables. According to commonly used judgment standards (19, 45), these VIFs are significantly <10, thereby indicating that the variables selected in this article do not have multicollinearity.

**Table 4:** Descriptive statistics and multicollinearity test.

**Variable**	**Obs**	**Mean**	**SD**	**Min**	**Max**	**VIF**
*self_HL*	18,069	3.049	1.025	1	5	–
*actual_HL*	19,259	3.076	0.763	1	4	–
*PITI*	8,658	58.89	12.41	21.65	82.40	1.02
*gender*	19,584	1.525	0.499	1	2	2.76
*Age*	19,262	62.00	10.12	45	118	1.22
*Edu*	19,584	3.448	1.938	1	11	1.28
*marriage*	19,597	0.849	0.358	0	1	1.12
*sleep*	19,661	6.302	2.135	0.5	15	1.03
*smoke*	19,597	0.427	0.495	0	1	2.43
*drinking*	19,501	2.400	0.874	1	3	1.35
*physic*	19,597	0.896	0.306	0	1	1.03
*insurance*	19,597	0.967	0.178	0	1	1.01
*health_ep*	18,933	6.986	24.77	0	1,200	1.01
*health_ch*	18,099	3.333	1.109	1	5	1.03
*urban*	19,597	0.403	0.491	0	1	1.11

### Ordered Logistic Regression Model

The explained variable in this paper is an ordinal variable, so it is not accurate enough to estimate with the traditional ordinary least square (OLS) regression model (46–48). The ordered logistic (Ologit) regression model is more suitable for dealing with such problems (49). It estimates the effects of a set of independent variables (numerical or categorical) on the logarithm of the probability that the dependent variable assumes low values rather than high values (50). The benchmark model constructed in this article is as follows:


$$\begin{array}{l} {HL}_{i}=\beta_{1}{PITI}_{i}+\beta_{2}{Control}_{i}+\varepsilon_{i} \end{array}$$

where *HL*_*i*_ is the health level of *i*, *PITI*_*i*_ is the PITI of the city where *i* is located, *Control*_*i*_ refers to all control variables, and ε_*i*_ is the random error term. Assuming that ε_*i*_ follows the logistic distribution, and the ordered explained variable has *j* distinct values, the relationship with the*X*_*k*_ explanatory variables for *j* varying between 1 and *J*-1 can be expressed through the following formula:


$$\text{log}\left[\frac{p\left(Y\leq \;\text{j}|X\right)}{\text{p}\left(Y>\;\text{j}|X\right)}\right]=\alpha_{\text{j}}-\sum_{\text{k}=1}^{\text{K}}{\text{β}}_{\text{k}}X_{\text{k}}=\alpha +X\text{β}$$

where α_*j*_ is the intercept and indicates the probability that the explained variable assumes low values rather than high values in case of nullity of all the explanatory variables. β_*k*_ represents the log(*ODDS*) change corresponding to a unitary increase of the *X*_*k*_ variables. Positive values of the β_*k*_ coefficients correspond to higher probabilities that the explained variable assumes high values, and vice versa (51).

## Results

### Tests of Parallel Regression Assumption

Ologit regression model is performed under the assumption of cumulative logit parallelity (52). Therefore, we need to test the parallel regression assumption of the model first, that is, to test whether the influence of each value level of the explanatory variable on the explained variable is the same in each regression equation. Table 5 shows the results of five test methods. The data in the table indicates that whether *self_HL* or *actual_HL* is used as the explained variable, and the *P*-value of the parallel hypothesis test obtained by all methods is >0.1, indicating that all the probabilities show none significance. In particular, the parallel regression assumption holds, and we can use the Ologit regression model for research.

**Table 5:** Parallel regression assumption test results.

**Test methods**	***self_HL***		***actual_HL***	
	**Chi2**	***P* > Chi2**	**Chi2**	***P* > Chi2**
Brant test	3.645	0.302	1.600	0.449
LR test	3.509	0.320	1.658	0.437
Wald test	3.493	0.322	1.659	0.436
Score test	3.494	0.322	1.659	0.436
Wolfe-Gould test	3.591	0.309	1.737	0.420

*H0: Parallel Regression Assumption holds*.

### Benchmark Regression

Table 6 shows the benchmark regression results of the model. Since the information reflected by the coefficients in the Ologit regression model is limited, we further calculate the odds ratio [*odds ratio* = exp(*coefficient*)] of each variable to make it easier to understand the regression results.

**Table 6:** Benchmark regression results.

**Variable**	***self_HL***		***actual_HL***	
	**Coefficient**	**Odds ratio**	**Coefficient**	**Odds ratio**
*PITI*	0.0099^***^	1.0100	0.0086^***^	1.0087
	(0.0018)		(0.0019)	
*gender*	−0.1665^**^	0.8464	−0.8318^***^	0.4353
	(0.0744)		(0.0788)	
*age*	−0.0221^***^	0.9787	−0.0628^***^	0.9392
	(0.0026)		(0.0027)	
*edu*	0.0391^***^	1.0399	0.1077^***^	1.1137
	(0.0129)		(0.0138)	
*marriage*	0.0495	1.0507	0.1408^*^	1.1512
	(0.0719)		(0.0734)	
*sleep*	0.1714^***^	1.1870	0.1354^***^	1.1450
	(0.0128)		(0.0136)	
*smoke*	−0.3001^***^	0.7408	−0.2983^***^	0.7421
	(0.0704)		(0.0745)	
*drinking*	−0.2521^***^	0.7771	−0.2460^***^	0.7819
	(0.0287)		(0.0306)	
*physic*	0.5765^***^	1.7798	1.0791^***^	2.9420
	(0.0975)		(0.0997)	
*insurance*	−0.1366	0.8723	−0.0797	0.9234
	(0.1447)		(0.1472)	
*health_ep*	−0.0066	0.9934	−0.0031^**^	0.9969
	(0.0042)		(0.0014)	
*health_ch*	0.1992^***^	1.2205	0.0573^***^	1.0589
	(0.0198)		(0.0215)	
*urban*	0.2613^***^	1.2986	0.1424^***^	1.1530
	(0.0474)		(0.0493)	
Pseudo *R*^2^	0.0434		0.1177	
*N*	7,533		7,533	

*Robust standard errors in parentheses*.

* *p < 0.1*,

** *p < 0.05*,

*** *p < 0.01*.

From the results in Table 6, we can conclude the following:

(1) Whether *self_HL* or *actual_HL* as the explained variable, the estimated coefficients of *PITI* are significantly positive, thereby indicating that environmental information disclosure has a significant positive correlation with the health level of middle-aged and old residents. When *self_HL* is used as the explained variable, the odds ratios of *PITI* is 1.0100, and when *actual_HL* is used as the explained variable, the odds ratios of *PITI* is 1.0087. These results indicate that when environmental information disclosure level increases by 1 unit, the probability of improving the self-reported health level and actual health level of middle-aged and old residents increases by 1 and 0.87%, respectively.
(2) In terms of the influence of the control variables on the explained variables, we choose a model with a better fitting effect for interpretation, that is, taking *actual_HL* as the explained variable. On the basis of the regression results, we can divide these control variables into three categories. The first type is that the regression coefficient is significantly positive, including *edu, marriage, sleep, physic, health_ch* and *urban*. Combined with Table 3, the outcome reveals that middle-aged and old residents with higher education and adequate sleep have better health conditions. Physical activity also has a significant positive impact on the health of middle-aged and old residents. Compared with rural areas, urban middle-aged and old residents have a higher level of health. The second type of control variable is that the regression coefficient is significantly negative, including *gender, age, smoke, drinking*, and *health_ep*. These results indicate that among middle-aged and old residents, the health of females is worse than that of males. In addition, the older the age, the higher the expenditure on medical and fitness, and the middle-aged and old residents who have smoking and drinking habits have worse health. The third type is the control variable with an insignificant regression coefficient, which is only *insurance*. The reason may be that this control variable in the sample does not have a significant binary classification. According to statistics, the medical insurance participation rate of Chinese residents in 2018 exceeded 95%.

### Robustness Test

#### Stepwise Regression

The process of stepwise regression is to first regress the explanatory variable to the explained variable separately and then gradually add the control variables and observe whether the sign and significance of the coefficient of the explanatory variable have changed to judge the robustness of the result. Tables 7, 8, respectively, show the stepwise regression results with *self_HL* and *actual_HL* as the explained variables. Among them, Column 1 presents the regression result without adding any control variables, and Columns 2–6 show the regression results after gradually adding the control variables. Owing to space limitations, we only list the coefficients, robust standard errors, and odds ratio of the explained variables in the text. The results in Tables 7, 8 show that whether *self_HL* or *actual_HL* is used as the explained variable, in all cases, the coefficient of the core explanatory variable *PITI* is significantly positive at the 1% statistical level, and the odds ratio is basically maintained at about 1%.

**Table 7:** Stepwise regression for *self_HL*.

**Variable**	**(1)**	**(2)**	**(3)**	**(4)**	**(5)**	**(6)**
*PITI*	0.0130^***^	0.0132^***^	0.0110^***^	0.0115^***^	0.0104^***^	0.0099^***^
	(0.0017)	(0.0017)	(0.0017)	(0.0018)	(0.0018)	(0.0018)
	[1.0130]	[1.0133]	[1.0110]	[1.0115]	[1.0104]	[1.0100]
Demographic characteristics	No	Yes	Yes	Yes	Yes	Yes
Living habits	No	No	Yes	Yes	Yes	Yes
Medical behavior	No	No	No	Yes	Yes	Yes
Health lever in childhood	No	No	No	No	Yes	Yes
Urban or rural residents	No	No	No	No	No	Yes
Pseudo *R*^2^	0.0027	0.0180	0.0348	0.0372	0.0418	0.0434
*N*	7,875	7,793	7,791	7,590	7,533	7,533

*Robust standard errors in parentheses. Odd ratio in brackets*.

^*^p < 0.1,

^**^p < 0.05,

*** *p < 0.01*.

**Table 8:** Stepwise Regression for *actual_HL*.

**Variable**	**(1)**	**(2)**	**(3)**	**(4)**	**(5)**	**(6)**
*PITI*	0.0091^***^	0.0111^***^	0.0096^***^	0.0096^***^	0.0088^***^	0.0086^***^
	(0.0017)	(0.0017)	(0.0018)	(0.0018)	(0.0019)	(0.0019)
	[1.0092]	[1.0111]	[1.0096]	[1.0096]	[1.0089]	[1.0087]
Demographic characteristics	No	Yes	Yes	Yes	Yes	Yes
Living habits	No	No	Yes	Yes	Yes	Yes
Medical behavior	No	No	No	Yes	Yes	Yes
Health lever in childhood	No	No	No	No	Yes	Yes
Urban or rural residents	No	No	No	No	No	Yes
Pseudo *R*^2^	0.0016	0.1009	0.1276	0.1267	0.1172	0.1177
*N*	8,429	8,313	8,313	8,089	7,533	7,533

*Robust standard errors in parentheses. Odd ratio in brackets*.

^*^p < 0.1,

^**^p < 0.05,

*** *p < 0.01*.

#### Change Estimation Method

Table 9 reports the estimated results using OLS and ordered probit regression models. We can find that after changing the estimation method, the regression coefficients of the core explanatory variable *PITI* to the explained variables *self_HL* and *actual_HL* are still significantly positive. This result confirms the robustness of the conclusions drawn in this article once more.

**Table 9:** Regression results of OLS and ordered probit models.

**Variable**	**OLS**		**Ordered probit**	
	***self_HL***	***actual_HL***	***self_HL***	***actual_HL***
*PITI*	0.0052^***^	0.0028^***^	0.0058^***^	0.0052^***^
	(0.0009)	(0.0006)	(0.0010)	(0.0011)
Control variables	Yes	Yes	Yes	Yes
*R*^2^/Pseudo *R*^2^	0.1045	0.2271	0.0419	0.1184
*N*	7,533	7,533	7,533	7,533

*Robust standard errors in parentheses*.

^*^p < 0.1,

^**^p < 0.05,

*** *p < 0.01*.

### Endogenous Problem Handling

There may be a reciprocal causation relationship between environmental information disclosure and the health of middle-aged and old residents. On the one hand, environmental information disclosure has an impact on the health of middle-aged and old residents, which is the causal relationship that this article focuses on. On the other hand, the poor health of middle-aged and old residents may force companies and governments to strengthen environmental information disclosure to promote environmental improvement. In addition, although we have added a large number of control variables to the model to minimize the impact of missing variables on the model estimation results, due to the limitations of the survey data and the complexity of the factors affecting the health of middle-aged and old residents, there may still be the problem of missing variables. All these will lead to endogenous problems in the model.

To solve the endogenous problem of the model, we choose the IV-Ordered probit regression model. With reference to Zhao (53), we select the lag period of PITI as an instrumental variable, including one lag period (*PITI*_*t*−1_) and three lag periods (*PITI*_*t*−3_). On the one hand, the level of public environmental information in cities generally has “inertial” characteristics, so the PITI in the lag period is significantly related to the PITI in the current period. On the other hand, the PITI in the lag period is difficult to directly impact the health of the current middle-aged and old residents.

Table 10 reports the regression results after dealing with the endogenous problem. From this table, we find that the Wald test of the two instrumental variables passed the 1% significance test, thereby rejecting the null hypothesis of weak instrumental variables. After the instrumental variables are added, the estimated coefficients of the core explanatory variable *PITI* for the explained variables *self_HL* and *actual_HL* are still significantly positive, reconfirming the conclusion that there is a significant positive relationship between environmental information disclosure and the health of middle-aged and old residents.

**Table 10:** Regression results of IV-Ordered probit.

**Variable**	**IV** **=** ***PITI***_***t−1***_		**IV** **=** ***PITI***_***t−3***_	
	***self_HL***	***actual_HL***	***self_HL***	***actual_HL***
*PITI*	0.0052^***^	0.0072^***^	0.0107^***^	0.0099^***^
	(0.0012)	(0.0013)	(0.0016)	(0.0017)
Control variables	Yes	Yes	Yes	Yes
Weak-IV: Wald test	18.57^***^	32.65^***^	44.23^***^	32.91^***^
*N*	8,658	8,658	8,658	8,658

*Robust standard errors in parentheses*.

^*^p < 0.1,

^**^p < 0.05,

*** *p < 0.01*.

### Regional Differences

To study whether there are regional differences in the impact of environmental information disclosure on the health of middle-aged and old residents, we divided all samples into three categories: eastern, central, and western. The eastern region includes Beijing, Tianjin, Hebei, Liaoning, Shanghai, Jiangsu, Zhejiang, Fujian, Shandong, Guangdong and Hainan. The central region includes Heilongjiang, Jilin, Shanxi, Anhui, Jiangxi, Henan, Hubei and Hunan. The western region includes Guizhou, Yunnan, Shaanxi, Inner Mongolia, Guangxi, Chongqing, Sichuan, Qinghai, Gansu, Ningxia and Xinjiang.

Table 11 shows the Ologit regression results of different regions. We select the explained variable *actual_HL* with better fitting effect. The results in Table 11 show that similar to the estimated results of the total sample, the estimated coefficients of *PITI* in different regions are all positive. However, only the estimated coefficients in the central region are significant, and the estimated coefficients in the eastern and western regions have not passed the significance test. This outcome shows that the impact of environmental information disclosure on the health of middle-aged and old residents is mainly manifested in the central region of China.

**Table 11:** Ologit regression results of different regions.

**Variable**	**Eastern region**	**Central region**	**Western region**
	***actual_HL***	***actual_HL***	***actual_HL***
*PITI*	0.0016	0.0269^***^	0.0071
	(0.0031)	(0.0054)	(0.0049)
Control variables	Yes	Yes	Yes
Pseudo *R*^2^	0.1191	0.1213	0.1242
*N*	3,303	2,124	2,106

*Robust standard errors in parentheses*.

^*^p < 0.1,

^**^p < 0.05,

*** *p < 0.01*.

## Discussion

(1) Forcing local governments to strengthen environmental management and improve the ecological environment is one of the roles of environmental information disclosure, which is conducive to improving the health of middle-aged and old residents. For a long time, China has been regarded as “information-poor”, and the country has strong control over economic development and information dissemination (54, 55). In the past 10 years, China's control of information and information processes has undergone major changes, especially in the field of environmental governance (56). The central government allows or even actively promotes the disclosure of environmental information and media reports to fight against powerful local polluters—usually some high-polluting manufacturing companies (34). However, local authorities generally do not support this environmental transparency because manufacturing companies are often an important source of tax revenue for local governments (57). With the openness of environmental information and the freedom of more media to report environmental disasters and protests, it is possible to resist the distortion of environmental information by local and regional authorities and force local governments to conduct environmental governance. The central government is likewise paying more and more attention to ecological and environmental issues in assessing the work and promotion of local officials, and the level of local environmental information disclosure is one of them (58). In the international community, The Chinese government has been calling on all countries to pursue innovative, coordinated, green and open development for all. The Chinese government has also been making its own contribution to controlling carbon emissions and addressing global climate change. Therefore, in the face of inspections and assessments from the central government and public supervision, local governments also form an accountability mechanism for environmental governance similar to the reverse effect (59). By increasing the transparency of environmental information, the central government, the general public, and the news media can strengthen the multiple accountabilities of local governments, thereby forcing them to strengthen environmental governance.
(2) Promoting companies to reduce pollution emissions and improve the ecological environment is another role of environmental information disclosure, which is also conducive to improving the health of middle-aged and old residents. Such disclosure can increase the environmental awareness of corporate management and stimulate changes in the production process (60). According to the theory of organizational legitimacy (61), pollutant companies are subject to pressure from regulatory agencies and public opinion when facing environmental information disclosure. If corporate behavior deviates from social values, then corporate legitimacy will be threatened and public policies that are unfavorable to corporate development will be incurred. To avoid these situations, companies will further strengthen technological upgrading and information disclosure, bear their due economic and social costs, reduce pollutant emissions, and improve their environmental efficiency. In addition to regulatory pressures, the need to improve financial performance also forces companies to take environmental protection actions (62). Environmental information disclosure can reduce information asymmetry between companies and investors, increase investors' trust in companies, and build social confidence (63, 64). It can also improve the company's green reputation (65). A good reputation can improve the company's relationship with stakeholders and attract more consumers (66), which will positively impact its financial performance.
(3) Environmental information disclosure is also an important driver of health behavior and decision-making changes among middle-aged and older residents. The first is by avoiding health risks. Passively perceiving changes in environmental pollution and strengthening environmental information disclosure can play certain roles in early warning. Since the physical health of middle-aged and old residents is more susceptible to threats than that of young people, as a rational individual, the former will quickly take measures against environmental pollution. For example, timely disclosure of air pollution information can enable middle-aged and old residents to reasonably plan outdoor activities based on environmental pollution state. Smog alarms issued through media channels, such as television, radio, and newspapers, can significantly reduce their daily attendance and outdoor activities (67, 68). The second is by actively participating in environmental protection. Public participation in environmental governance must be based on a certain amount of environmental information; however, the public will pay a lot of private costs, such as time and money, to collect relevant environmental information (69). Environmental information disclosure can allow middle-aged and old residents to understand the surrounding environmental conditions and existing problems, point out the direction for participating in environmental governance, and reduce the difficulty of their participation in environmental governance. Such disclosure can improve the scientific understanding of environmental issues and ecological protection awareness of middle-aged and old residents (70). At the same time, it helps them increase their trust in the government, thereby increasing their enthusiasm to participate in environmental governance. Furthermore, in the field of public health, non-government actors such as private sectors, social society, philanthropy, academia play their role to improve the health of all. The implementation of the environmental information disclosure system also helps to enhance their motivation and convenience to participate in environmental governance. The third is by rising health care expenditures. A study based on data from Indian households shows that households who are told their drinking water may be contaminated will increase their spending on water purification (71). The environmental information disclosure system can also bring the same effect. It can inform middle-aged and old residents whether the surrounding environmental pollution is severe. When environmental pollution is serious, middle-aged and old residents increase their health care expenditures to protect their health, such as purchasing protective equipment, sports equipment, and health care drugs.
(4) The impact of environmental information disclosure on the health of middle-aged and old residents also has significant regional heterogeneity. Specifically, the impact is mainly reflected in the central region of China. The reason for this result may be derived from the high pollution in central China. Compared with the eastern and western regions, the central region of China has a large labor force and low labor costs. Meanwhile, the cost of land in the central region is lower, and there are more relaxed environmental regulations. Enterprises with high pollution are more willing to build factories in the central region. Therefore, the central region has become the most concentrated area of environmental pollution in China and is facing more severe environmental pollution challenges. Official statistics from the Chinese government show that in terms of waste water, waste gas and industrial solid waste, the central region has significantly higher emissions than the eastern and western regions, with Shanxi and Anhui provinces experiencing particularly severe pollution. Enhancing the disclosure of environmental information can restrain the pollutant discharge behavior of polluting enterprises and promote the process of environmental pollution control in the central region. Therefore, the health level of middle-aged and old residents in the central region is more prominently affected by environmental information disclosure.

## Conclusion

Based on the data of PITI and CHARLS in China, this study uses the Ologit regression model to explore the impact of environmental information disclosure on the health of middle-aged and old residents. Results show that environmental information disclosure can improve the health of middle-aged and old residents. After the robustness test and endogenous problem handling, this conclusion still holds. Estimation results of the Ologit regression model show that when PITI increases by 1 unit, the probability of improving the self-reported health level and actual health level of middle-aged and old residents increases by 1 and 0.87%, respectively. In addition, due to different levels of environmental pollution, the impact of environmental information disclosure on the health of middle-aged and old residents has significant regional heterogeneity. Specifically, the impact is mainly reflected in the central region of China.

In order to improve the health level of middle-aged and old residents, we suggest that the Chinese government should further improve the environmental information disclosure system, and take the reception rate and satisfaction rate of residents as important indicators to evaluate the implementation effect of the environmental information disclosure system. Meanwhile, the government should timely adjust the content and form of environmental information disclosure according to the needs of residents. In addition, the government also needs to formulate different environmental information disclosure goals according to the actual characteristics of environmental pollution in different regions.

According to the classification of the three actors of government, enterprises, and individuals, we discuss the specific mechanism of this effect and present in three paths: forcing local governments to govern the environment, promoting enterprises to reduce pollution, and influencing individuals to make behavioral decisions. However, we did not test these mechanisms with mathematical models, which is also the specific direction for our future research.

## Data Availability Statement

Publicly available datasets were analyzed in this study. This data can be found here: Data of CHARLS-2018 are available at http://charls.pku.edu.cn/, and data of PITI are available at http://www.ipe.org.cn/.

## Author Contributions

XY and WS: conceptualization and data collection. XY: analyzed data and writing—original draft preparation. XY and SL: writing—review and editing. All authors contributed to the article and approved the submitted version.

## Funding

This research was funded by the Natural Science Foundation of Zhejiang (No. LQ22G030002), the National Social Science Fund of China, (No. 19AZD004), the National Natural Science Foundation of China (No. 71874092), and K.C. Wong Magna Fund in Ningbo University.

## Conflict of Interest

The authors declare that the research was conducted in the absence of any commercial or financial relationships that could be construed as a potential conflict of interest.

## Publisher's Note

All claims expressed in this article are solely those of the authors and do not necessarily represent those of their affiliated organizations, or those of the publisher, the editors and the reviewers. Any product that may be evaluated in this article, or claim that may be made by its manufacturer, is not guaranteed or endorsed by the publisher.
